# Domain Requirements and Genetic Interactions of the Mud1 Subunit of the *Saccharomyces cerevisiae* U1 snRNP

**DOI:** 10.1534/g3.118.200781

**Published:** 2018-11-09

**Authors:** Radhika Agarwal, Beate Schwer, Stewart Shuman

**Affiliations:** * Molecular Biology Program, Sloan-Kettering Institute, New York, NY 10065; † Microbiology and Immunology Department, Weill Cornell Medical College, New York, NY 10065

**Keywords:** pre-mRNA splicing, U1 snRNP, RRM domains, mutagenesis, synthetic lethality

## Abstract

Mud1 is an inessential 298-amino acid protein subunit of the *Saccharomyces cerevisiae* U1 snRNP. Mud1 consists of N-terminal and C-terminal RRM domains (RRM1 and RRM2) separated by a linker domain. Synthetic lethal interactions of *mud1*∆ with deletions of inessential spliceosome components Nam8, Mud2, and Msl1, or missense mutations in the branchpoint-binding protein Msl5 enabled us to dissect genetically the domain requirements for Mud1 function. We find that the biological activities of Mud1 can be complemented by co-expressing separately the RRM1 (aa 1-127) and linker-RRM2 (aa 128-298) modules. Whereas RRM1 and RRM2 (aa 197-298) *per se* are inactive in all tests of functional complementation, the linker-RRM2 by itself partially complements a subset of synthetic lethal *mud1*∆ interactions. Linker segment aa 155 to 196 contains a nuclear localization signal rich in basic amino acids that is necessary for RRM2 activity in *mud1*∆ complementation. Alanine scanning mutagenesis indicates that none of the individual RRM1 amino acid contacts to U1 snRNA in the cryo-EM model of the yeast U1 snRNP is necessary for *mud1*∆ complementation activity.

pre-mRNA splicing initiates when the U1 snRNP engages the intron 5′ splice site (5′SS). The *Saccharomyces cerevisiae* U1 snRNP consists of a trimethylguanosine (TMG) capped 568-nt U1 snRNA, a seven-subunit Sm protein ring (present also in the U2, U4, and U5 snRNPs), and ten U1-specific protein subunits: Prp39, Prp40, Snu71, Snu56, Snp1, Mud1, Luc7, Prp42, Nam8, and Yhc1 (Gottschalk *et al.* 1998; Fortes *et al.* 1999a; Schwer *et al.* 2011). Base-pairing of the U1 snRNA leader motif 5′-ACUUAC sequence with the consensus yeast 5′SS element 5′-GUAUGU nucleates an initial U1•pre-mRNA complex. Cross-intron bridging interactions between the yeast U1 snRNP at the 5′SS and the Msl5•Mud2 heterodimer at the branchpoint sequence 5′-UACUAAC then stabilize a commitment complex, which provides a scaffold for recruitment of the U2 snRNP to the branchpoint (Abovich and Rosbash 1997).

Traditional genetics and synthetic genetic arraying, as well as structure-guided mutagenesis, have identified a rich network of genetically buffered functions during early spliceosome assembly in budding yeast, embracing the U1-specific snRNP proteins Mud1, Nam8, Yhc1, Snp1, and Luc7, the U1 snRNA, the TMG cap, the Cbc2•Sto1 nuclear m^7^G cap-binding complex (CBC), the DEAD-box ATPase Prp28, the Msl5•Mud2 branchpoint-binding complex, the Msl1 and Lea1 subunits of the U2 snRNP, and the seven subunits of the Sm protein ring (Liao *et al.* 1993; Abovich *et al.* 1994; Colot *et al.* 1996; Gottschalk *et al.* 1998; Staley and Guthrie 1999; Fortes *et al.* 1999b; Chen *et al.* 2001; Hausmann *et al.* 2008; Wilmes *et al.* 2008; Hage *et al.* 2009; Costanzo *et al.* 2010; Qiu *et al.* 2012; 2015; Chang *et al.* 2012; Schwer *et al.* 2013; 2016; 2017; Schwer and Shuman 2014; 2015; Jacewicz *et al.* 2014; 2015; Agarwal *et al.* 2016). This network is defined by the numerous instances in which null alleles of inessential players (*e.g.*, Mud1, Nam8, Mud2) or benign mutations in essential factors (*e.g.*, Yhc1, Snp1, Luc7, Msl5, Sm ring subunits) elicit synthetic lethal phenotypes when combined with other benign mutations in the splicing machinery.

The composition of the U1 snRNP is more complex in budding yeast than in mammals, with respect to the size of the U1 snRNA (568 *vs.* 164 nt) and the number of U1-specific protein subunits (ten *vs.* three) that are assimilated into U1 snRNP along with the 7-subunit Sm protein ring. Yeast Snp1, Mud1, and Yhc1 are the homologs of human U1-70K, U1A, and U1C, respectively. Whereas Snp1 and Yhc1 are essential for yeast viability, Mud1 is not (Smith and Barrell 1991; Kao and Siliciano 1992; Liao *et al.* 1993; Tang *et al.* 1997). This contrasts with the essentiality of Mud1 homolog U1A for mammalian U1 snRNP function. U1A and Mud1 consist of two RRM (RNA recognition motif) domains separated by a linker peptide. U1A has been the subject of intense study as a paradigm of RNA recognition by RRM-containing proteins (reviewed in Maris *et al.* 2005). Indeed, the landmark crystal structure of the N-terminal RRM1 domain of U1A bound to the stem-loop II segment in human U1 snRNA (Oubridge *et al.* 1994) established key principles that apply broadly to the RRM family. U1A RRM1 specifically recognizes the sequence AUUGCAC atop stem-loop II in the mammalian U1 RNA. By contrast, the U1A C-terminal RRM2 domain does not bind RNA (Lu and Hall 1997).

Mud1 does not recapitulate U1A in the respective yeast and human U1 snRNPs, insofar as the yeast U1 snRNA does not have an equivalent of the human U1 snRNA stem-loop II element to which U1A binds. Tang and Rosbash (1996) employed *in vivo* dimethylsulfate (DMS) modification to probe the U1 snRNA in *MUD1 vs. mud1*∆ cells and thereby presented evidence for enhanced DMS accessibility to two regions of yeast U1 snRNA (nt 61 to 68 and 133 to 150, imputed to comprise opposing looped out segments within a duplex stem) when Mud1 is absent. As neither of these regions has any sequence similarity to the AUUGCAC site recognized by U1A, one of the following scenarios can be surmised: (i) if Mud1 binds yeast U1 snRNA, it does so with different specificity than U1A; (ii) Mud1 does not bind U1 snRNA directly and the DMS accessibility absent Mud1 reflects indirect effects on U1 snRNA conformation or on protein interaction within the U1 snRNP. Whereas Tang and Rosbash (1996) showed that deleting RRM2 of Mud1 had no effect on DMS sensitivity (implying that RRM2 is not essential for association of Mud1 with U1 snRNP), the ∆RRM2 version of Mud1 was biologically inactive in an *in vivo* reporter assay employing an inefficiently spliced synthetic intron that depends on Mud1. They proposed that RRM1 serves to tether RRM2 to the U1 snRNP.

Here we revisit the issue of how the domains of Mud1 are organized functionally. We exploit an expanded array of synthetic genetic interactions of Mud1 uncovered in the two decades since Mud1 was last studied. By surveying for complementation of synthetic lethality by Mud1 domains, *per se* and in various combinations, we show that RRM1 and RRM2 are both necessary for full Mud1 biological activity, though they need not be linked within the same polypeptide. Specifically, we find that co-expression of an RRM1-containing N-terminal fragment Mud1-(1-127) plus a C-terminal fragment Mud1-(128-298), comprising an interdomain linker and RRM2, was able to complement *mud1*∆ synthetic lethality in multiple genetic backgrounds. The linker segment, which we show includes a nuclear localization signal (NLS), was necessary to confer activity on RRM2. Guided by a recent cryo-EM structure of yeast U1 snRNP (Li *et al.* 2017), we conducted an alanine scan of RRM1 amino acids that contact the U1 snRNA and found that none were essential *per se* for Mud1 activity *in vivo*.

## MATERIALS AND METHODS

### MUD1 expression plasmids and mutants

Yeast plasmids pRS415-MUD1 (*CEN LEU2*) and pRS413-MUD1 (*CEN HIS3*) contain a *MUD1* expression cassette composed of the *MUD1* cDNA, 569-bp of 5′-flanking genomic DNA (containing the putative *MUD1* promoter), and 309-bp of 3′-flanking genomic DNA. Restriction sites for *Sac*I and *Xma*I were introduced at the 5′ and 3′ ends of the cassette to allow insertion between the *Sac*I and *Xma*I sites of the pRS415 and pRS413 plasmids. A *Bam*HI site was introduced immediately 5′ of the *MUD1* translation start codon and a *Spe*I site was placed immediately 3′ of the stop codon; these sites facilitated cloning of *MUD1* mutant alleles. (We observed no difference in the growth of yeast strains expressing the *MUD1* cDNA compared to a strain expressing the intron-containing *MUD1* gene.) N-terminal truncation variants were generated by PCR using forward primers that introduced a *Bam*HI site followed by a Met residue in lieu of Lys127, Ile137, Lys147, Ala157, Lys177, and Glu196. C-terminal truncation variants were generated by PCR using reverse primers that introduced stop codons and a flanking *Spe*I site in lieu of Gly128 and Asn197. Alanine mutations were introduced into the full-length *MUD1* expression cassette by two-stage PCR overlap extension with mutagenic primers.

Plasmids for co-expression of Mud1 domain fragments (under the control of the *MUD1* promoter) were constructed as follows. First, the expression cassettes for N-terminal truncations *MUD1-(128-298)* and *MUD1-(197-298)* were PCR amplified from the pRS413 plasmids using a forward primer that introduced an *Xma*I site 569-bp upstream of the start codon (to replace the *Sac*I site) and a reverse primer that introduced an *Apa*I site 309-bp downstream of the stop codon (to replace the *Xma*I site). The PCR products were digested with *Xma*I and *Apa*I and inserted between *Xma*I and *Apa*I sites of p415-MUD1-(1-127) and p415-MUD1-(1-196) to generate plasmids p415-MUD1-(1-127+128-298), p415-MUD1-(1-196+197-298), and p415-MUD1-(1-127+197-298) with the two *MUD1* alleles arranged in a head-to-tail configuration. The *MUD1* ORFs in the expression plasmids were sequenced completely to confirm that no unwanted changes were acquired during amplification and cloning.

### Tests of Mud1 function in vivo

We employed plasmid shuffle assays to test the effects of Mud1 mutations on complementation of *mud1*∆ synthetic lethality with *mud2*∆, *nam8*∆, *MSL5-L169A*, *MSL5-I189A*, and *MSL5-Y100A* (Chang *et al.* 2010; Qiu *et al.* 2011; Jacewicz *et al.* 2015). The yeast strains used for plasmid shuffle are listed in Table S1. To establish the complementation assay in the *msl1*∆ background, we prepared a heterozygous *MSL1msl1*::*natMX MUD1mud1*::*kanMX* diploid by crossing a *msl1*::*natMX* haploid (generated by integration of a *natMX* cassette flanked by 500 bp and 464 bp segments of genomic DNA upstream and downstream of the *MSL1* ORF) with *mud1*::*kanMX* cells of the opposite mating type. The diploids were then transformed with plasmid p360-MUD1 [*CEN URA3MUD1*]. Ura^+^ diploids were selected and sporulated. Asci were dissected and the desired haploid *mud1*∆ *msl1*∆ progeny were Ura^+^ and resistant to clonNAT and geneticin. When *mud1*∆ *msl1*∆ p360-MUD1 cells were transferred to agar medium containing 5-fluoroorotic acid (FOA) to select against the *URA3MUD1* plasmid, only tiny colonies were recovered after 6 days of incubation at 30°. The resulting *mud1*∆ *msl1*∆ strain failed to form colonies on YPD agar (Fig. S1).

To assay bioactivity of wild-type and mutated *MUD1* alleles, the double-mutant strains were transfected with *CEN LEU2MUD1* plasmids. In case of *mud1*∆ *msl5*∆, *MUD1* alleles on *CEN LEU2* plasmids were co-transfected with *MSL5-Ala* alleles on *CEN HIS3* plasmids. Transformants were selected and streaked on agar medium containing FOA. The plates were incubated at 20, 30, and 37°, and alleles that failed to give rise to macroscopic colonies at any temperature after 8 days were deemed lethal. Individual FOA-resistant colonies with viable *MUD1* alleles were grown to mid-log phase in YPD broth and adjusted to *A*_600_ of 0.1. Aliquots (3 µl) of serial 10-fold dilutions were spotted on YPD agar plates, which were then incubated at temperatures ranging from 20 to 37°.

### Mud1 linker-GFP reporters of cellular localization

Two-stage PCR overlap extension was used to create DNA fragments in which the Mud1 linker segments aa 128-196, aa 128-154, and aa 155-196 were fused in-frame to the N-terminus of green fluorescent protein (GFP). pYN132-GFP (Schneider and Schwer 2001) was used as the template to amplify the GFP open reading frame. In the process, restriction sites were introduced for cloning of the DNA fragments into a pRS423-based plasmid (*2µ HIS3*) in which expression of GFP and the linker-GFP fusions is under the transcriptional control of the *TPI1* promoter. The resulting constructs were transfected into *W303a* cells and transformants were selected on SD-His agar. Individual colonies were inoculated into SD-His liquid medium and grown at 30° to an *A*_600_ of 0.6. Aliquots of the cultures were harvested by centrifugation and resuspended in 4% paraformaldehyde for fixation. After washing with phosphate buffered saline (PBS), the cells were stained with DAPI. The cells were then washed again with PBS and mounted to a glass slide. Images were captured using an Axio2 microscope (Zeiss) with a 100x objective connected to a charge-coupled device camera and processed using SlideBook software (Intelligent Imaging Innovations).

### Data availability

Strains and plasmids are available upon request. The authors affirm that all data necessary for confirming the conclusions of the article are present within the article, figures, and tables. Supplemental material available at Figshare: https://doi.org/10.25387/g3.7291493.

## RESULTS and DISCUSSION

### Complementation of synthetic lethal mud1∆ phenotypes by expression of Mud1 domains

**Figure 1:**
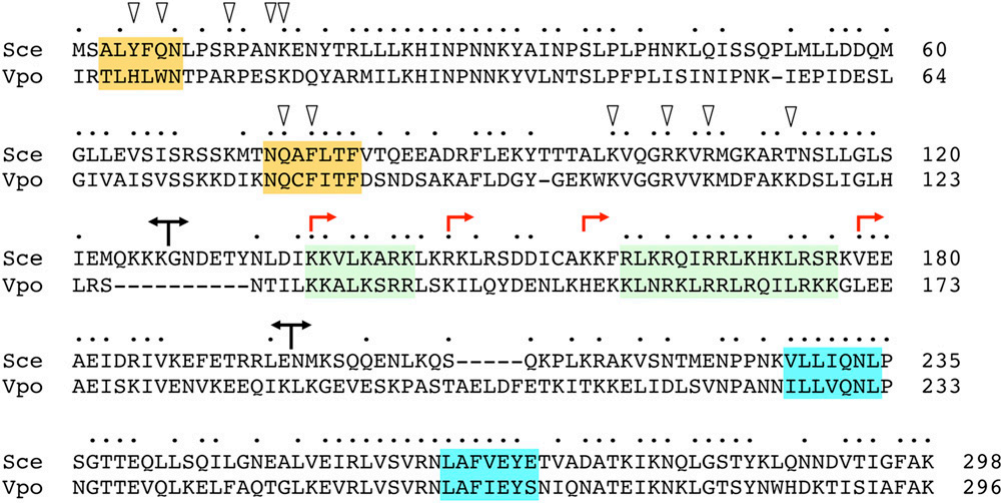
*Saccharomomyces cerevisiae* Mud1. The primary structures of the Mud1 polypeptides of *Saccharomyces cerevisiae* (Sce) and *Vanderwaltozyma polyspora* (Vpo) are aligned. Positions of side chain identity/similarity are indicated by •. Gaps in the alignment are denoted by dashes. Forward and reverse arrowheads indicate the boundaries of *S. cerevisiae* Mud1 N-terminal and C-terminal truncations, respectively, that were generated in the present study. The putative RNP motifs of the RRM1 and RRM2 domains are shaded gold and blue, respectively. Two blocks of basic amino acids in the linker are shaded green. The Mud1 amino acids subjected to alanine mutagenesis here are indicated by triangles.

**Figure 2:**
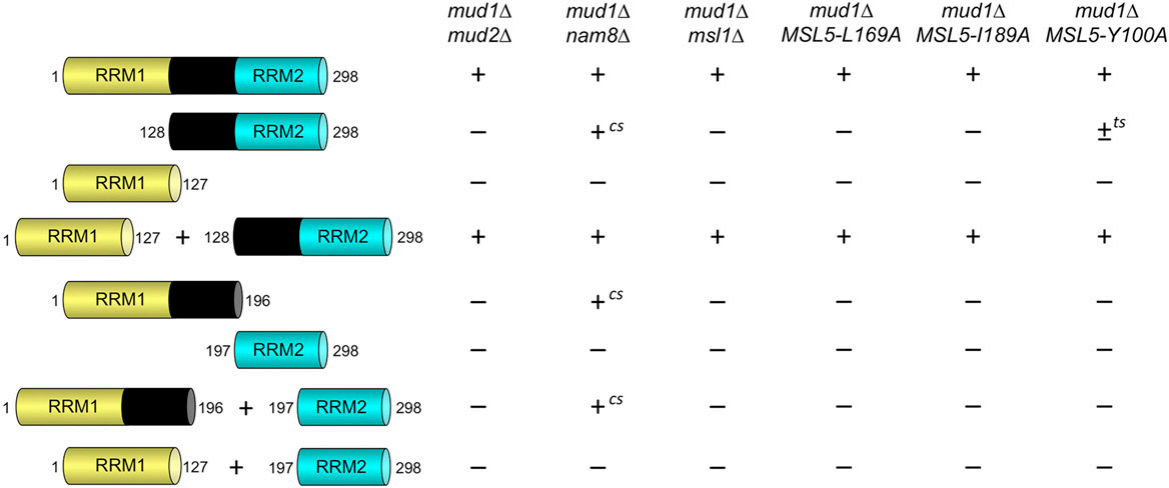
Mud1 domain requirements in multiple synthetic lethal genetic backgrounds. Wild-type and truncated *MUD1* alleles encoding the indicated Mud1 polypeptides were tested for activity by plasmid shuffle in six different double-mutant genetic backgrounds in which *MUD1* deletion is lethal. *MUD1* alleles, and combinations thereof, that failed to support growth of the test strains on FOA were deemed lethal (scored as – growth). The viable FOA-resistant strains expressing the indicated Mud1 proteins were spot-tested for growth on YPD agar at temperatures from 20°C to 37°C. Growth was scored as follows: (+) colony size indistinguishable from strains bearing wild-type *MUD1*; (+*^cs^*) + growth at 37°C and no growth at 20°C; (±*^ts^*) no growth at 37°C and small colonies at other temperatures.

Yeast Mud1 and human U1A share an RRM1-linker-RRM2 domain organization, but are not highly similar at the amino acid sequence level. By contrast, a primary structure alignment of the 298-aa *S. cerevisiae* Mud1 protein to the homologous 296-aa protein from the fungus *Vanderwaltozyma polyspora* highlights 167 positions of side chain identity/similarity (Figure 1). We used this alignment as a guide to divide Mud1 into the following segments: RRM1 (aa 1-127); linker-RRM2 (aa 128-298); RRM1-linker (aa 1-196); and RRM2 (aa 197-298). *CEN* plasmids bearing wild-type *MUD1* or truncated *MUD1* alleles under the control of the native *MUD1* promoter were tested by plasmid shuffle for complementation of *mud1*∆ in six different double-mutant genetic backgrounds in which *mud1*∆ is synthetically lethal. These included: (i) absence of U1 snRNP subunit Nam8 (Qiu *et al.* 2011), branchpoint binding protein subunit Mud2 (Abovich *et al.* 1994; Chang *et al.* 2010), and U2 snRNP subunit Msl1 (Fig. S1); and (ii) alanine mutations of Msl5 within the intron branchpoint RNA-binding domain (L169A, I189A) or in the Mud2-interaction domain (Y100A) (Schwer *et al.* 2013). Expression of full-length wild-type Mud1 complemented the lethality of all test strains. Expression of RRM1 or RRM2 alone failed to provide Mud1 function in any genetic background tested (Figure 2). Restoring the linker segment to RRM2 elicited a gain of partial function, whereby linker-RRM2 expression weakly complemented *mud1*∆ in the context of *MSL5-Y100A* (scored as ± and temperature-sensitive *ts*) (Figure 2) and *nam8*∆ (+ growth and cold-sensitive; *cs*) (Figures 2 and S2). Appending the linker to RRM1 allowed complementation of *nam8*∆, albeit not at low temperature (Figure 2 and Figure S2).

The salient finding was that co-expression of RRM1 and linker-RRM2 as separate proteins complemented Mud1 function in all six synthetic lethal genetic backgrounds (Figure 2). Thus, whereas the RRM1 and RRM2 domains are both required for Mud1 activity, they need not be linked in *cis* within the same polypeptide. Additional insight was gained from the observations that: (i) co-expression of RRM1 and RRM2 (*i.e.*, devoid of linker) failed to support growth of the six test strains; and (ii) co-expressing RRM1-linker and RRM2 was no better than expressing RRM1-linker alone (Figure 2). These results indicate that the linker is needed in *cis* for activity of the RRM2 domain.

### Probing the requirements for linker domain function

**Figure 3:**
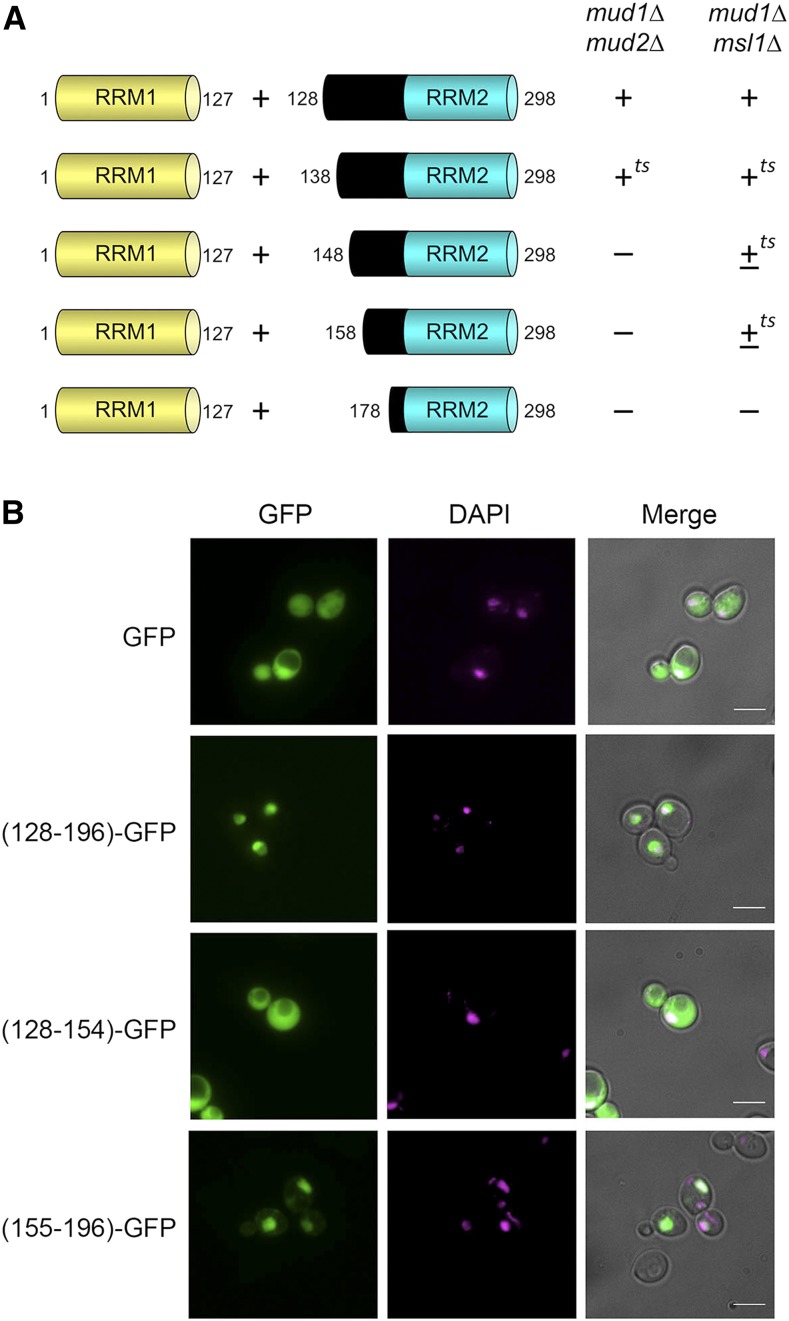
Function of the Mud1 linker region. (A) The indicated linker-RRM2 proteins plus RRM1 were co-expressed and tested by plasmid shuffle for complementation of *mud1*∆ *mud2*∆ and *mud1*∆ *msl1*∆ strains. Combinations that failed to support growth of the test strains on FOA were deemed lethal (scored as –). The viable FOA-resistant strains expressing the indicated Mud1 proteins were spot-tested for growth on YPD agar at temperatures from 20°C to 37°C. Growth was scored as follows: (+) colony size indistinguishable from strains bearing wild-type *MUD1*; (+*^ts^*) + growth at 20 and 25°C and no growth at 37°C; (±*^ts^*) no growth at 37°C and small colonies at other temperatures. (B) The linker acts as a nuclear localization signal (NLS). The linker region and its C- and N- terminal truncation*s* were fused to the N-terminus of GFP. These fusion proteins were expressed in *W303a* cells. GFP (green) was detected by fluorescence microscopy. The nuclei (magenta) were visualized with DAPI. The scale bar denotes 5 µm.

The Mud1 linker domain is rich in arginines and lysines (25/69 positions; Figure 1), which are distributed in several clusters suggestive of a nuclear localization signal (NLS). Indeed, the linker domain of mammalian U1A is also arginine-lysine rich and has been shown to function as a long and complex NLS for U1A (Kambach and Mattaj 1992; Hetzer and Mattaj 2000; Hieda *et al.* 2001). To begin to delineate the important features of the linker domain we serially truncated the original linker-RRM2 protein (aa 128-298) to generate Mud1 constructs 138-298, 148-298, 158-298, and 178-298 (Figure 1, N-termini indicated by red arrows). These alleles were co-expressed with RRM1 and tested for complementation in two genetic backgrounds in which the linker was found to be essential for RRM2 activity: *mud1*∆ *mud2*∆ and *mud1*∆ *msl1*∆. Expression of the Mud1-(138-298) protein in tandem with RRM1 sustained growth of *mud1*∆ *mud2*∆ and *mud1*∆ *msl1*∆ cells at 20 to 30° but not at 37° (scored as +*^ts^* in Figure 3A). The Mud1-(148-298), -(158-298), and -(178-298) constructs failed to complement *mud1*∆ *mud2*∆ when co-expressed with RRM1, signifying that the Mud1 segment from aa 138 to 147 is essential in this genetic background. By contrast, Mud1-(148-298) or Mud1-(158-298) plus RRM1 complemented the *mud1*∆ *msl1*∆ strain and conferred a slow growth phenotype at 20 to 30° and no growth at 37° (scored as ±*^ts^* in Figure 3A). This result underscores that the linker requirements vary in different genetic contexts. A further deletion of the linker segment from aa 158 to 177 abolished *mud1*∆ *msl1*∆ complementation (Figure 3A).

To test if the linker can function as an NLS, we created a series of plasmid-borne Mud1 linker-GFP reporters in which the linker segments from aa 128-196, 128-154, or 155-196 were fused to the N-terminus of green fluorescent protein (GFP). These linker-GFP plasmids, and a control plasmid expressing GFP, were introduced into wild-type yeast cells and the distribution of GFP was gauged by fluorescence microscopy. Whereas the GFP control was distributed throughout the yeast cell (exclusive of the vacuole), the (128-196)-GFP fusion was concentrated in the nucleus (Figure 3B). Upon bisecting the linker in the fusion construct, we saw that whereas (128-154)-GFP was distributed diffusely (*à la* the GFP control), (155-196)-GFP localized to the nucleus (Figure 3B). We conclude that an NLS resides within the Mud1 linker segment from aa 155-196, likely by virtue of the basic patch ^161^RLKRQIRRLKHKLRSR^176^ that is conserved in *V. polyspora* Mud1 (Figure 1). We infer that the necessity of this segment for Mud1 function in complementing *mud1*∆ synthetic lethality (Figure 3A) reflects its capacity to direct the Mud1 RRM2 to the nucleus wherein splicing occurs.

### Alanine scanning mutagenesis of Mud1 RRM1

**Figure 4:**
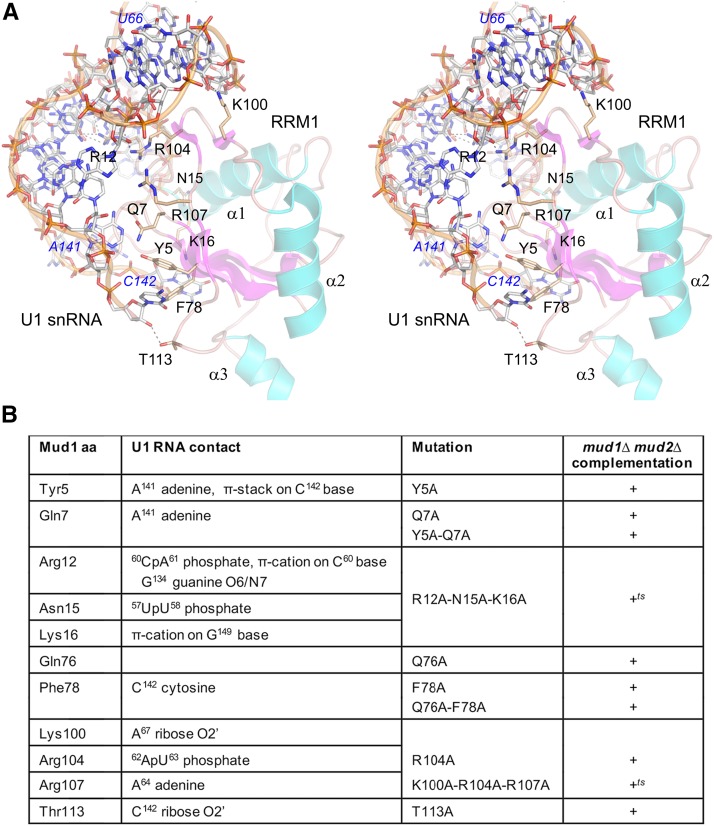
Alanine scanning mutagenesis of Mud1. (A) Stereo view of the modeled Mud1 RRM1 domain and its interaction with U1 snRNA, prepared in Pymol using data from the cryo-EM structure in pdb 5UZ5. Mud1 is depicted as a cartoon trace with magenta β strands and cyan α helices. Amino acids subjected to alanine scanning are rendered as stick models with beige carbons. The segment of U1 snRNA with which Mud1 interacts is depicted as a stick model with gray carbons and a gold cartoon trace through the phosphodiester backbone. (B) Summary of the mutational analysis. Mud1 amino acids and their U1 snRNA contacts are listed in the first two columns on the left. Single, double, or triple alanine mutants of full-length Mud1 (listed in the third column) were tested by plasmid shuffle for *mud1*∆ *mud2*∆ complementation (fourth column at right).

We undertook alanine scanning mutagenesis of *S. cerevisiae* Mud1 RRM1, guided initially by primary structure similarity to *V. polyspora* Mud1 (Figure 1) and other fungal homologs and by presumptive similarities to the RRM1 component of U1A (Oubridge *et al.* 1994), the goal being to probe the effects of mutations at the putative Mud1•U1 snRNA interface. The subsequent report of the cryo-EM structure of the *S. cerevisiae* U1 snRNP (Li *et al.* 2017) allowed us to focus the findings we report here on mutations of Mud1 amino acids that interact with the U1 snRNA. The deposited U1 snRNP model (pdb 5UZ5) is missing large segments of many of the component proteins; in the case of Mud1, the model includes segments from aa 3 to 45, 55 to 125, and 132 to 148 (Li *et al.* 2017), *i.e.*, there is no modeled structure of RRM2 or the essential portion of the linker (or the TAP-tag on the Mud1 C-terminus that was used by Li *et al.* to affinity-purify the U1 snRNP). The cryo-EM model of Mud1 RRM1 comprises a four-strand anti-parallel β sheet, a β hairpin loop, and three α helices (Figure 4A). RRM1 contacts the U1 snRNA segments from nucleotides U^57^ to U^66^, C^140^ to C^142^, and G^149^ to G^150^ (Figure 4A). It is especially noteworthy that these Mud1 contacts to U1 snRNA in the EM structure model correspond to the sites of enhanced DMS modification of the U1 snRNA in *mud1*∆ cells that were identified by Tang and Rosbash (1996) as the putative U1 snRNA-binding sites for Mud1 RRM1.

Here we introduced alanine substitutions at ten Mud1 amino acids that contact the U1 snRNA: Tyr5, Gln7, Arg12, Asn15, Lys16, Phe78, Lys100, Arg104, Arg107, and Thr113. Their contacts are indicated in Figure 4B and illustrated in a stereo view of the RRM1-RNA interface in Figure 4A. We also introduced alanine at Gln76 in the RNP1 motif of RRM1 (**Q**AFLTF; shaded gold in Figure 1); this side chain is conserved in the RNP1 motif of *V. polyspora* Mud1 (**Q**CFITF) and human U1A (**Q**AFVIF). The human U1A Gln side chain makes a π stacking interaction with purine nucleobase G9 of human U1 snRNA (Oubridge *et al.* 1994). The Mud1 Gln76 side chain is not modeled beyond the β-carbon in the yeast U1 snRNP structure. Single, double, and triple alanine mutations were introduced into the full-length *MUD1* gene and the mutant alleles were assayed by plasmid shuffle for *mud1*∆ *mud2*∆ complementation (Figure 4B). All six of the single-alanine mutants – *Y5A*, *Q7A*, *R104A*, *T113A*, *Q76A*, and *F78A* – were as effective as wild-type *MUD1* in supporting growth of *mud1*∆ *mud2*∆ cells at 20 to 37°, as were the *Y5A-Q7A* and *Q76A-F78A* double mutants (Figure 4B). *mud1*∆ *mud2*∆ cells complemented by triple mutants *R12A-N15A-K16A* and *K100A-R014A-R107A* grew well at 20 to 34° but formed small colonies at 37° (scored as +*^ts^* in Figure 4B).

### Concluding remarks

The present study affords new genetic insights into Mud1 domain organization and structure-activity relations. By taking advantage of multiple genetic backgrounds in which Mud1 is essential for yeast vegetative growth, we delineated three Mud1 domains – RRM1, linker, and RRM2 – that are organized as two functional units: RRM1 and linker-RRM2, respectively. Tang and Rosbash (1996) were prescient in their deductions from DMS footprinting of the sites of contact between Mud1 and the U1 snRNA and their attribution of U1 snRNA contacts to the RRM1 domain, which apparently sufficed to be recruited to the U1 snRNP in the absence of RRM2. The key finding here is that RRM1 and linker-RRM2 need not be linked in *cis* within the same polypeptide to fulfill all Mud1 functions for vegetative growth, *i.e.*, co-expression of RRM1 and linker-RRM2 as separate polypeptides complemented *mud1*∆ in every strain background. This belies the model suggested initially that RRM1 serves to tether the RRM2 to the U1 snRNP. Instead, our data indicate that the linker domain, which is needed in *cis* for activity of the RRM2 domain, directs RRM2 to the U1 snRNP independent of RRM1. The linker segment immediately flanking RRM2 contains an NLS that can guide the linker-RRM2 unit to the nucleus. The linker domain might also deliver RRM2 to the U1 snRNP, *e.g.*, by virtue of interactions of the tracts of basic amino acids in the linker (Figure 1) with U1 snRNA or other protein subunits of the U1 snRNP. The linker segment ^132^TYNLDIKKVLKARKLKR^148^ that embraces the upstream basic tract is modeled in cryo-EM structures of the U1 snRNP and the *S. cerevisiae* pre-spliceosome as a long α helix (Li *et al.* 2017; Plaschka *et al.* 2018; Bai *et al.* 2018). However, this α helix is not in direct contact with the U1 snRNA and is remote from other protein subunits. The disposition of the linker NLS segment and RRM2 within the U1 snRNP is presently uncharted.

Differences in the ability of domain fragments of Mud1 to complement the growth of six synthetically lethal *mud1*∆ strains suggest a contextual hierarchy of Mud1 necessity ranging from: (i) most exigent, as in the cases of *mud1*∆ *mud2*∆, *mud1*∆ *msl1*∆, *mud1*∆ *MSL5-L169A*, and *mud1*∆ *MSL5-I189A* in which only the combination of RRM1 and linker-RRM2 sustains growth; to (ii) less stringent, exemplified by *mud1*∆ *nam8∆*, in which either RRM1-linker or linker-RRM2 alone can restore viability, albeit with *cs* phenotypes (Figure 2).

We conclude from the structure-guided alanine scan of the RRM1 domain that: (i) none of the individual Mud1 RRM1 amino acid contacts to U1 snRNA in the cryo-EM model is necessary for Mud1 activity in an exigent genetic background (*mud1*∆ *mud2*∆); (ii) loss of both contacts of Tyr5 and Gln7 to the A141 adenine is benign; and (iii) triple mutations that eliminate multiple RNA contacts (*e.g.*, to nucleotides U^57^ to A^61^ or A^62^ to A^67^) are viable albeit *ts*.

The results here, and the recent structural studies of the yeast U1 snRNP, underscore differences between yeast Mud1 and human U1A, to the point that they might best be viewed as structural homologs (*i.e.*, composed of two RRMs separated by a linker) but not as functional orthologs, notwithstanding a shared affiliation with their respective U1 snRNPs. The salient point is that Mud1 and U1A recognize entirely different sites in the yeast and human U1 snRNAs (Oubridge *et al.* 1994; Li *et al.* 2017; Plaschka *et al.* 2018; Bai *et al.* 2018).
